# Postoperative cognitive dysfunction in elderly postcardiac surgery patients: progress in rehabilitation application research

**DOI:** 10.3389/fresc.2024.1525813

**Published:** 2024-12-17

**Authors:** Zhen-Rong Zhang, Yang-Zheng Li, Xiao-Qing Wu, Wen-Jun Chen, Jian Xu, Wei-Hua Zhao, Xiao-Yan Gong

**Affiliations:** ^1^ Department of Rehabilitation Medicine, Sir Run Run Shaw Hospital, School of Medicine, Zhejiang University, Hangzhou, Zhejiang, China; ^2^ Department of Rehabilitation Medicine, The First People’s Hospital of Shizuishan, Shizuishan, Ningxia, China; ^3^ Nursing Department, Sir Run Run Shaw Hospital, School of Medicine, Zhejiang University, Hangzhou, Zhejiang, China

**Keywords:** postoperative cognitive dysfunction, risk factors, cardiac surgery, rehabilitation assessment, rehabilitation therapy

## Abstract

Postoperative cognitive dysfunction (POCD) is a prevalent complication of the central nervous system in elderly patients following cardiac surgery. This review aims to provide an overview of the etiology, risk factors, diagnostic assessment, and rehabilitation strategies for cognitive dysfunction occurring after cardiac surgery. The pathogenesis of POCD after cardiac surgery includes cerebral microembolism, neuroinflammation, and cryptogenic strokes. Risk factors are associated with advanced age, diminished preoperative cognitive status, and anesthesia. Cognitive function screening tools used for pre- and postoperative assessments can detect changes in patients’ cognitive levels in a timely manner. The timely provision of appropriate rehabilitation methods, including cognitive function training, exercise training, transcranial direct current stimulation, and perioperative acupuncture, is crucial, with emerging technologies such as virtual reality playing an increasingly significant role. In conclusion, POCD is a common postoperative complication in elderly cardiac surgery patients, with age and reduced preoperative cognitive function being the primary risk factors. A comprehensive rehabilitation strategy can more effectively address postoperative cognitive dysfunction in patients.

## 1 Introduction

Postoperative cognitive dysfunction (POCD) refers to perioperative disorders of attention, memory, thinking, logic, mental activity, or sleep during the perioperative period, which can be categorized according to the time of onset as follows: (1) cognitive dysfunction that already presents prior to surgery; (2) postoperative delirium (POD), neuropsychiatric dysfunction occurring within 1 week postoperatively or prior to discharge; (3) delayed neurocognitive recovery, cognitive dysfunction existing within 30 days after surgery; (4) postoperative neurocognitive impairment, cognitive dysfunction existing from 30 days to 12 months after surgery; and (5) cognitive decompensation, first diagnosed 12 months after surgery (1, 2). Compared with preoperative levels, POCD manifests itself as a decline in cognitive function after surgery and anesthesia. In addition to impaired learning and memory and poor concentration, there may even be alterations in personality, social skills, and cognitive abilities, which can be extremely distressing for patients (3, 4).

PCOD is a common complication after cardiac surgery in elderly individuals, with a prevalence ranging from 9% to 54%, and the highest incidence is observed after open aortic, transcatheter aortic valve implantation and coronary artery bypass graft (CABG) surgery (5). With the development of society and the advancement of medical technology, the number of elderly patients has increased, and the perioperative and in-hospital mortality rates for surgical patients have decreased significantly (6). However, these patients are at a much greater risk of decreased overall cognitive function, long-term cognitive decline, reduced quality of life, and increased hospitalization costs and mortality than nonelderly patients are (3, 7, 8). Compared with noncardiac surgeries, the incidence of POCD is more pronounced in cardiac surgery patients, necessitating heightened vigilance. Furthermore, the cognitive impairments that arise following cardiac surgery may be more severe and could be correlated with the progression to dementia (9). Relander et al. reported that immediate postoperative cognitive decline, notably in executive function, in 71% of cardiac surgery patients significantly predicts long-term cognitive deterioration (10). These findings may be attributed to the intricacy of the surgical procedure, the application of extracorporeal circulation techniques during surgery, extended operative durations, and the inherent cardiovascular risk factors for the patient (11).

Currently, the main method of POCD intervention is prevention by controlling or eliminating modifiable risk factors, which fails to summarize the rehabilitation assessment and rehabilitation strategies for patients with cognitive dysfunction and is unable to carry out targeted prevention and intervention for high-risk patients. Therefore, systematic and comprehensive management of postoperative patients through a series of scientific and standardized measures is particularly important. This review summarizes the pathogenesis, risk factors, diagnostic assessment, and intervention strategies for POCD after cardiac surgery to improve the overall understanding of POCD among healthcare teams and ultimately improve the long-term quality of life of patients.

## 2 Pathogenesis

The underlying pathophysiology of POCD after cardiac surgery is still unknown, and several existing mechanisms, including neuroinflammation, cerebral microembolism, and cryptogenic stroke, are known.

### 2.1 Cerebral microembolism

After the intraoperative application of conventional extracorporeal circulation, neuropsychological deficits in patients undergoing CABG surgery are associated with the number of microemboli generated during the procedure (12). Abu-Omar Y's study demonstrated that the number of cerebral microemboli was significantly greater in patients who underwent extracorporeal circulation than in those who did not, and functional MRI revealed a significant relative reduction in prefrontal activation, which may be related to subclinical dysfunction (13). De Carlo et al. reported that the development of silent cerebral ischemic lesions was associated with more pronounced transient neurocognitive decline early in the postoperative period after transcatheter aortic valve replacement and a lower rate of recovery at follow-up, which suggests that the development of cerebral microembolism has an impact on neurocognitive function (14). The number of cerebral microemboli can be reduced by a noncorporeal route or by shortening the duration of extracorporeal circulation, although no reduction in the incidence of short-term postoperative cognitive dysfunction has been observed (15, 16).

### 2.2 Neuroinflammation

Surgical stimulation and necrotic tissue from surgical trauma can activate the immune system and release a variety of proinflammatory factors, causing a nonspecific inflammatory response in the body (17, 18). However, an excessive inflammatory response may damage the body's own normal tissues. On the one hand, some inflammatory factors may disrupt the blood‒brain barrier and thus be transported into the central nervous system (CNS), thereby triggering neurogenic neuroinflammation (19). On the other hand, proinflammatory factors reach the microcirculation of the CNS along with the blood circulation, bind to the corresponding receptors, and activate the corresponding immune cells to produce new proinflammatory factors in the CNS (20). Inflammatory factors damage key components of cellular mitochondria, such as nucleic acids and proteins, by mediating oxidative and nitrative stress responses, resulting in mitochondrial damage; impaired energy metabolism in the oxidative respiratory chain; and ultimately necrosis, apoptosis, or degenerative changes in CNS cells, which are key components of neuroinflammation-induced neuronal cell injury (21, 22). Correspondingly, Taylor et al. found that the level of serum interleukin-6 in elderly patients undergoing major surgery is correlated with their executive function within one year after surgery (23). Correlative imaging examinations revealed significantly reduced seed-to-voxel functional connectivity of the left dorsolateral prefrontal cortex with the superior parietal lobe and diminished negative connectivity in the default mode network, including the angular gyrus and posterior cingulate gyrus, in patients who underwent cardiac surgery 7 days later (24).

### 2.3 Cryptogenic strokes

Cryptogenic strokes are more common than overt strokes in nonoperative patients and are associated with cognitive decline. Cryptogenic strokes may enhance abnormalities associated with Alzheimer's disease by triggering the development of senile plaques and neurofibrillary tangles, reflecting the brain fragility or certain vascular risk characteristics (25, 26). Although asymptomatic cerebrovascular disease is an important cause of aging, little is known about perioperative cryptogenic stroke in existing research (27). A study of perioperative cryptogenic stroke in patients undergoing noncardiac surgery revealed that perioperative cryptogenic stroke occurred in 1 in 14 patients older than 65 years who underwent noncardiac surgery and was associated with an increased risk of cognitive decline at 1 year (28). Another international multicenter study revealed that the incidence of perioperative cryptogenic stroke was as high as 10.0% in patients ≥65 years of age who underwent noncardiac surgery (29). This aspect is more prevalent among patients undergoing cardiac surgery, with approximately 18% having a history of stroke or transient ischemic attack (30). In a prospective study by Browne et al. of 49 patients who underwent CABG, perioperative cryptogenic strokes occurred in 19 (39%) patients, POD occurred in 5 (26%) patients with cryptogenic strokes, and POD occurred in 3 (10%) patients who did not experience cryptogenic strokes (31). Although sophisticated neuroimaging techniques can discern early and subtle damage within acute ischemic lesions, validated predictive biomarkers to identify patients at elevated risk for perioperative cryptogenic stroke are lacking. The future necessitates the prompt identification of patients with higher risk profiles, coupled with an intensified investigation into preventative and therapeutic approaches concerning perioperative cryptogenic stroke.

## 3 Risk factors and predictors

For patients undergoing noncardiac surgery, POCD is usually the result of a synergistic effect of multiple factors, including but not limited to, advanced age; declining cognitive reserve; lower levels of education; diabetes mellitus; alcoholism; sensory impairments; malnutrition; increased duration of anesthesia; secondary surgeries; postoperative infections; respiratory complications; and psychological factors (32–34). Intraoperative hypoxemia and/or hypocapnia are dose-dependently associated with a greater risk of POD, which may have an impact on patients’ long-term cognitive function after surgery (35).

Among elderly patients undergoing CABG or percutaneous coronary intervention, levels of long-term postoperative cognitive function decline more rapidly than they do in the general population, regardless of whether they receive extracorporeal circulation, but no difference in the degree of memory decline was found between patients who underwent these two procedures (36). Xie et al. created a column chart by analyzing preoperative and intraoperative data from cardiac patients and reported that combining patients’ cardiopulmonary bypass time, hypertension, white blood cell count, aspartate aminotransferase, and arrhythmia results could be a good predictor of the risk of cardiac POCD, with good results in internal validation (37). There are also observational studies suggesting that natural aging may have a greater impact on patients’ long-term cognitive dysfunction than surgery does. Selnes et al. followed 152 patients who underwent CABG surgery and 92 nonsurgical cardiac patients for 6 years and reported mild cognitive decline, but the difference was not statistically significant (38). van Dijk et al. reported the same results after a 5-year-long follow-up study of 281 patients who underwent CABG surgery and 112 healthy subjects without coronary artery disease, with no statistically significant difference in cognitive decline between the two groups (39). POD in cardiac surgery patients, potentially influenced by factors such as age, diabetes, preoperative depression, mild cognitive impairment, carotid artery stenosis, NYHA functional class III or IV, mechanical ventilation duration, and ICU stay, may predispose patients to the development of POCD (32).

The effects of several factors, such as hypotension (34, 40) and intraoperative hyperoxia (7, 41), on POCD are highly controversial, and the role of these factors in the occurrence of POCD is not yet clear. However, considering the large methodological differences among related studies, more high-quality studies are still needed to explore the associations between these factors and POCD.

## 4 Evaluations and assessment

Routine preoperative assessment of cognitive function in elderly patients is needed to evaluate their preoperative cognitive functional status, which can help stratify the risk of developing POCD in patients to adjust the subsequent preventive, monitoring, and therapeutic strategies. Patients with preoperative cognitive impairment and dementia should be further evaluated for activities of daily living (ADLs) and psychobehavioral symptoms, and neuropsychological testing, laboratory tests, and imaging should be performed as necessary. Repeat cognitive screening or neuropsychological testing after surgery can help identify new cognitive impairments (Table 1).

**Table 1:** Rehabilitation assessment strategies for perioperative cognitive dysfunction.

Preoperative screening	Further examinations	Diagnosis
MMSE	Neuropsychological tests: memory, visuospatial structure, language, psychomotor speed and attention/concentration	DSM-5
MoCA	ADLs	ICD-11
Mini-Cog	Laboratory tests: biomarker tests	
CDR	Imaging tests: MRI, CT	
GDS	Genetic testing	
Number games		
EEG		

CDR: Clinical Dementia Rating Scale; CT: Computed Tomography; DSM-5: Diagnostic and Statistical Manual of Mental Disorders, 5th Edition; EEG: Electroencephalography; GDS: Global Deterioration Scale; ICD-11: International Classification of Diseases and Related Health Problems, 11th Edition; MMSE: Minimum Mental State Examination; MoCA: Montreal Cognitive Assessment; MRI: Nuclear Magnetic Resonance Imaging.

### 4.1 Preoperative cognitive function assessment

The Minimum Mental State Examination (MMSE) is one of the most influential and popular international screening tools for cognitive dysfunction, with tests covering time and place orientation, immediate memory, attention, numeracy, short-term memory, language, and visuospatial structural skills (42). The Montreal Cognitive Assessment (MoCA) covers a broader range of cognitive domains than the MMSE does, including attention, executive functioning, memory, language, visuospatial structural skills, abstract thinking, numeracy, and orientation, and its use for mild cognitive dysfunction screening is more accurate than the MMSE (43). Preoperative screening for cognitive function can also be performed via the Brief Mental Status Assessment Scale (Mini-Cog), which involves a 3-item word recall test for memory, and the clock drawing test as a distractor, which tests visuospatial presentation, recall, and executive functioning (44). The Clinical Dementia Rating Scale (CDR) is a standardized tool for assessing the severity of dementia symptoms; it helps doctors and researchers obtain a more accurate picture of a patient's cognitive functioning and is particularly widely used in the clinical diagnosis and research of dementia (45). The general decline scale (GDS), developed by Dr. Barry Reisberg, divides a patient's cognitive decline into seven stages, focusing on signs and symptoms that reflect the later stages of dementia (46).

In addition, a number of new assessments have begun to emerge that, when standardized and regulated, might be applied to the preoperative assessment of cognitive function. For example, Lucatelli et al. and Goulart et al. used the number game of the MentalPlus® test and reported that this method was effective in monitoring preoperative and postoperative cognitive decline in patients and had a moderate-to-strong correlation with standardized tests assessing short-term memory and visual perception (9, 47). Geraedts et al. reported the use of preoperative EEG-based machine learning technology to predict cognitive decline after deep brain stimulation of the thalamus in patients with Parkinson's disease and that cortical neurophysiological alterations could be used as biomarkers during screening (48).

### 4.2 Further examination of preoperative cognitive dysfunction

For patients with mild cognitive impairment and dementia, further testing of ADLs, assessment of psychobehavioral symptoms (e.g., anxiety, depression, etc.), tests of cognitive functioning in specific domains (e.g., memory, visuospatial structure, language, psychomotor speed, and attention/concentration), and, if necessary, further biomarkers (e.g., amyloid, etc.) and imaging (e.g., MRI and CT) may be indicated.

### 4.3 Diagnosis of new-onset POCD

POCD is diagnosed via the DSM-5 criteria for mild/severe neurocognitive impairment, as evidenced by mild/significant cognitive decline in 1 or more cognitive domains (complex attention, executive functioning, learning and memory, language, perceptual-motor, or social-cognitive) compared with levels of prior performance (1). Mild neurocognitive dysfunction is defined as a reduction in cognitive function scores of 1–2 standard deviations from baseline values or controls and cognitive deficits that do not interfere with independence in daily activities; severe neurocognitive dysfunction is defined as a reduction in cognitive function scores of more than 2 standard deviations or more from baseline values or controls and cognitive deficits that interfere with independence in daily activities (49). Patients can also be diagnosed with cognitive dysfunction by referring to the Eleventh Revision of the Mental and Behavioral Disorders chapter of the International Classification of Diseases and Related Health Problems, 11th edition (ICD-11) by the World Health Organization (50).

## 5 Comprehensive cognitive rehabilitation strategies

For patients with preoperative combined cognitive decline, in addition to basic clinical treatment, cognitive rehabilitation, including cognitive function training, improved nutritional status and increased exercise training, is needed. Reasonable choices of anesthetic drugs and anesthetic methods during surgery and intraoperative temperature management, circulatory management and respiratory management can also reduce the occurrence of POCD (51). For high-risk patients, increasing the depth of anesthesia and cerebral oxygen saturation monitoring can be considered to reduce brain damage caused by too deep anesthesia or too low cerebral oxygen saturation (52). Early postoperative monitoring of changes in the patient's level of cognitive function is also needed to make timely adjustments to medications and the patient's physical status (53). In addition, the integrated use of multiple rehabilitation training methods, such as cognitive function training, exercise training, transcranial direct current stimulation (tDCS), perioperative acupuncture, transcranial magnetic stimulation (TMS), virtual reality (VR) technology and other therapeutic methods, can better prevent or improve the cognitive decline of patients (Table 2).

**Table 2:** Comprehensive rehabilitation strategies for perioperative cognitive dysfunction.

Preoperative management	Intraoperative management	Postoperative rehabilitation
Risk factor assessment	Appropriate anesthesia drugs and methods	Perioperative cognitive training
Prehabilitation to improve preoperative cognition	Depth of anesthesia monitoring	Exercise training
Improvement of nutritional status and physical condition	Temperature management	TMS
Exercise training	Circulatory management	tDCS
	Respiratory management	Acupuncture
	Acupuncture	VR

tDCS: transcranial Direct Current Stimulation; TMS: Transcranial Magnetic Stimulation; VR: Virtual Reality.

### 5.1 Cognitive training

Preoperative cognitive optimization can be implemented in any setting and may have a positive impact on the prognosis of elderly surgical patients (54). A randomized clinical trial conducted by O'Gara et al. in cardiac surgery patients revealed that adherence to postoperative training in cardiac surgery patients could be improved by cognitive prehabilitation designed in the preoperative period, which may lead to an improvement in postoperative cognitive rehabilitation outcomes (55). Another study by Humeidan et al. revealed that cognitive prehabilitation did not reduce the incidence of POD in elderly patients undergoing major, noncardiac, nonneurological surgery under general anesthesia (56). Related studies have not yet been able to reveal the impact of preoperative prehabilitation on patients’ long-term cognitive functioning or the optimal cognitive training modality and training dose.

Perioperative cognitive training has been shown to be effective in postoperative cognitive rehabilitation. Butz et al. reported that postoperative cardiac patients had a significantly lower risk of cognitive dysfunction after 3 weeks of cognitive rehabilitation than did those who did not receive cognitive rehabilitation at discharge or 3 months postdischarge (57). Song et al. conducted an 8-week home computerized cognitive training program for elderly postlung transplant patients and reported that patients in the intervention group performed better than did those in the control group on both the verbal fluency test and the forward digit memory breadth test (58). A meta-analysis conducted by Li et al. also demonstrated that perioperative cognitive training was effective in decreasing the incidence of POCD, although no reduction in the incidence of POD could be observed (59). Further work is needed to determine the effectiveness of cognitive rehabilitation in preventing and improving POD and POCD, as well as to determine the optimal duration and frequency of treatment for perioperative cognitive dysfunction training.

### 5.2 Exercise training

Appropriate perioperative exercise training maintains optimal physical condition and thus reduces the risk of developing perioperative neurocognitive deficits (60). ten Brinke et al. conducted an 8-week computerized cognitive training program in elderly healthy individuals and reported that exercise training prior to cognitive training improved subjects’ executive function better (61). A randomized controlled trial conducted by Ji et al. also revealed that exercise training improved patients’ executive function and physical status, but when exercise training was combined with cognitive training, it improved patients’ physical performance, especially dynamic balance (62). Exercise training may improve cognitive function in patients by enhancing mitochondrial stability and energy metabolism, promoting CNS neuroplasticity (63). These findings suggest that exercise training can improve the preoperative cognitive function and physical condition of surgical patients, thus preventing POCD.

Different exercise training modalities may have different effects on patients’ cognitive function recovery. A meta-analysis found that resistance training, in isolation or when combined with aerobic training, may lead to greater improvements in physical and functional recovery following cardiac surgery via median sternotomy (64). Another recent clinical trial has yielded similar results, showing that 12 week early moderate intensity resistance training is more effective in improving cognitive function in patients after cardiac surgery compared to standard aerospace based rehabilitation (65). However, due to the limitation of sample size, further research is needed to determine the dosage of exercise training needed to determine the optimal exercise modalities, intensities, durations, and frequencies, among other parameters.

### 5.3 Transcranial direct current stimulation

tDCS relieves symptoms; improves cognitive performance caused by neuropsychiatric disorders; and enhances cognitive abilities, including memory, attention and perception (66, 67). Relevant basic studies have shown that anodic tDCS in the right frontal region can modulate effective connectivity and synchronization between different regions of the brain, including the frontal cortex, parietal cortex, and thalamus, and thus shorten the duration of delirium symptoms in rats after microelectrode implantation (68). A randomized controlled trial by Tao et al. involving a single session of anodic tDCS in the left dorsal and lateral prefrontal cortex in elderly patients who had undergone major lower limb arthroplasty reported a reduction in POD (69). Although tDCS has not been used in long-term POCD, this noninvasive brain stimulation technique may also be part of future preventive alternative therapies for cognitive dysfunction.

### 5.4 Transcranial magnetic stimulation

TMS improves cognitive function by enhancing neuroplasticity, which helps the brain form new neural connections (70). Different patterns of repetitive TMS can be used to modulate neural activity, resulting in a significant increase in the connectivity and reorganization of brain networks, leading to improved cognitive performance (71, 72). TMS has been shown to have efficacy in many disorders affecting cognitive function, such as stroke (59), Alzheimer's disease (73), brain injury and multiple sclerosis (74), but its use in the cognitive function of surgical patients has not been reported. Compared with conventional TMS, theta-burst stimulation can modulate cortical excitability by simulating the endogenous theta rhythm of the human body and emitting a series of short and rapid combinations of magnetic pulses, which can be used with shorter stimulation times and lower stimulation intensities (75). A related study on the use of theta-burst stimulation in elderly postoperative orthopedic patients is recruiting subjects and assessing the severity and duration of POD, cognitive function, pain, and performance of ADLs, among other relevant indicators (76). It should be noted that there is currently no direct evidence of the effectiveness of TMS in patients with POCD, with the exception of one safety and proof-of-concept study of patients who underwent surgical removal of brain tumors (77).

### 5.5 Perioperative acupuncture

The mechanism by which perioperative acupuncture protects cognitive function remains unclear, but research suggests that it may include inhibiting neuroinflammation, suppressing levels of oxidative stress, reducing neuronal damage, enhancing synaptic plasticity, and modulating the microbiota brain‒gut axis (78, 79). Preoperative acupuncture can alleviate anxiety and optimize the preoperative state (80). Preoperative acupuncture can reduce the use of anesthetics and analgesics; alleviate anesthesia-related side effects; help stabilize respiratory and circulatory functions; and protect the heart, brain and other important organs (79, 81). Postoperative acupuncture can help relieve pain, shorten hospital stays, and improve the long-term patient prognosis (82). As acupuncture research continues to expand, the mechanism of action and long-term health status of postoperative patients need to be clarified as soon as possible to better guide the clinical treatment of POCD patients, and more high-quality research on optimal treatment protocols is needed.

### 5.6 Novel cognitive training methods based on VR and other pathways

VR and artificial intelligence technologies, such as artificial intelligence assistance and deep learning, can be used to conduct cognitive training through the construction of 3D scenarios (e.g., supermarkets, kitchens, and neighborhoods) to increase patients’ interest and level of engagement. VR interventions have two unique characteristics, immersion and interactivity, which are considered mechanisms for beneficial effects. Immersion can bring about a sense of embodiment and elicit genuine physiological and psychological responses (83). Interactivity allows for real-time feedback and has a reward system that increases the motivation of the participants and improves compliance, treatment endurance, and happiness (84). A meta-analysis of VR interventions to improve mild cognitive dysfunction revealed that VR interventions used for cognitive rehabilitation improved patients’ cognitive functioning (e.g., memory, dual-tasking, and visual attention) and psychological functioning (e.g., reduced anxiety, increased well-being, and increased use of coping strategies) (85). Another meta-analysis similarly revealed that VR improved cognitive and motor function in older adults with mild cognitive impairment or dementia, particularly in the areas of attention/execution, memory, overall cognition and balance (86). Although no evidence that VR improves short-term cognitive dysfunction in postoperative patients has been reported, VR may affect patients’ pain perception, which in turn may lead to changes in their long-term prognosis (87). Furthermore, combining VR interventions with exercise and cognitive training could be a good option for people with POCD or dementia (86). The pilot program for the relevant study has been published, but the results are still being collected (88).

## 6 Summary and outlook

At present, several challenges persist in the rehabilitation and assessment of geriatric patients following cardiac surgery. First, most existing studies have focused on patients without preoperative cognitive impairment, and it is unclear whether perioperative cognitive rehabilitation improves postoperative outcomes and cognitive function in patients with preoperative cognitive problems. Second, most interventions only assess the occurrence of short-term POD and cognitive function, and the long-term prognosis of patients and long-term cognitive level changes need to be assessed in the future. Finally, it is necessary to improve more accurate screening and evaluation strategies for elderly patients undergoing cardiac surgery, in order to develop appropriate combination of rehabilitation program and the most beneficial treatment methods and doses for patients with POCD.

In the context of the growing emphasis on perioperative care, better recovery and long-term quality of life have become paramount concerns. The integration of multiple rehabilitation strategies during the perioperative period may yield more substantial benefits. For example, the combination of exercise and cognitive training may significantly enhance postoperative cognitive function (89). The widespread use of telephone and video conferencing provides a simpler method for monitoring and improving the long-term prognosis of patients. By developing a remote cognitive screening and training system, it is expected to simplify the preoperative and postoperative evaluation process, thereby enabling the prompt commencement of cognitive rehabilitation initiatives designed to oversee and refine the long-term trajectory of cognitive faculties. These advancements provide a more efficacious and variegated spectrum of alternatives for postoperative cognitive dysfunction rehabilitation evaluation and training in cardiac patients.
